# Involvement of Small Non-Coding RNA and Cell Antigens in Pathogenesis of Extramedullary Multiple Myeloma

**DOI:** 10.3390/ijms232314765

**Published:** 2022-11-25

**Authors:** Monika Vlachová, Jana Gregorová, Petra Vychytilová-Faltejsková, Natalia Anna Gabło, Lenka Radová, Lenka Pospíšilová, Martina Almáši, Martin Štork, Zdeňka Knechtová, Jiří Minařík, Tereza Popková, Tomáš Jelínek, Roman Hájek, Luděk Pour, Lucie Říhová, Sabina Ševčíková

**Affiliations:** 1 Babak Myeloma Group, Department of Pathophysiology, Faculty of Medicine, Masaryk University, 625 00 Brno, Czech Republic; 2 Centre for Molecular Medicine, Central European Institute of Technology, Masaryk University, 625 00 Brno, Czech Republic; 3 Institute of Biostatistics and Analyses, Faculty of Medicine, Masaryk University, 625 00 Brno, Czech Republic; 4 Department of Clinical Hematology, University Hospital Brno, 625 00 Brno, Czech Republic; 5 Department of Internal Medicine, Hematology and Oncology, University Hospital Brno, 625 00 Brno, Czech Republic; 6 Department of Hemato-Oncology, University Hospital Olomouc and Faculty of Medicine and Dentistry, Palacky University Olomouc, 779 00 Olomouc, Czech Republic; 7 Department of Hematooncology, University Hospital Ostrava, Ostrava, and Faculty of Medicine, University Ostrava, 708 00 Ostrava, Czech Republic

**Keywords:** multiple myeloma, NGS, microRNA, immunophenotyping, bioinformatics

## Abstract

Extramedullary multiple myeloma (EMD) is an aggressive disease; malignant plasma cells lose their dependence in the bone marrow microenvironment and migrate into tissues. EMD is a negative prognostic factor of survival. Using flow cytometry and next-generation sequencing, we aimed to identify antigens and microRNAs (miRNAs) involved in EMD pathogenesis. Flow cytometry analysis revealed significant differences in the level of clonal plasma cells between MM and EMD patients, while the expression of CD markers was comparable between these two groups. Further, miR-26a-5p and miR-30e-5p were found to be significantly down-regulated in EMD compared to MM. Based on the expression of miR-26a-5p, we were able to distinguish these two groups of patients with high sensitivity and specificity. In addition, the involvement of deregulated miRNAs in cell cycle regulation, ubiquitin-mediated proteolysis and signaling pathways associated with infections or neurological disorders was observed using GO and KEGG pathways enrichment analysis. Subsequently, a correlation between the expression of analyzed miRNAs and the levels of CD molecules was observed. Finally, clinicopathological characteristics as well as CD antigens associated with the prognosis of MM and EMD patients were identified. Altogether, we identified several molecules possibly involved in the transformation of MM into EMD.

## 1. Introduction

Multiple myeloma (MM) is the second most common hematological malignancy characterized by a proliferation of malignant plasma cells (PCs) that accumulate in the bone marrow (BM) and displace normal hematopoiesis. These malignant PCs produce monoclonal immunoglobulin detectable in the serum and/or urine of MM patients [1]. In the Czech Republic, MM incidence was reported at 4.8/100,000 per year; the median age at diagnosis was 65 years [2].

Previously, the definition of MM was based on the so-called CRAB symptoms (hyperCalcemia, Renal failure, Anemia and Bone lesions) corresponding to end-organ damage [3]. The introduction of new drugs (immunomodulatory drugs, proteasome inhibitors and monoclonal antibodies) dramatically increased the survival of MM patients; MM diagnosis was redefined by shifting treatment to earlier stages. Currently, MM is defined based on the presence of clonal bone marrow plasma cells >10% or biopsy-proven plasmocytoma and the presence of at least one myeloma-defining event (evidence of end-organ damage or at least one marker of malignancy (clonal bone marrow plasma cells >60%)) involved: uninvolved serum-free light chains ratio >100 or more than 1 focal lesion on MRI [4].

Unfortunately, a longer survival of MM patients has been connected to reports about an increased incidence of so-called extramedullary MM (extramedullary disease, EMD) [5,6,7,8]. In EMD, a subclone of PCs loses its dependence on the BM microenvironment and leaves the BM microenvironment, sometimes forming distinct tumors. EMD is associated with swift disease progression, poor prognosis and even treatment resistance [9,10].

Two subtypes of EMD have been described—primary EMD (present at the time of MM diagnosis) and secondary EMD (present at the time of MM relapse). Moreover, EMD may be divided into two subgroups: bone-related EMD (EMD-B) and soft-tissue-related EMD (EMD-S). While EMD-B is still partially dependent on the BM microenvironment, EMD-S is completely independent [6,7,11]. The underlying pathogenesis of MM to EMD progression has not been completely clarified.

Most human genes are regulated by microRNAs (miRNAs), which are short, highly conserved non-coding RNA molecules that regulate various cellular processes [12]. These non-coding RNA molecules are also significantly involved in tumorigenesis [13,14,15,16]. As previously described, so-called ‘hallmarks of cancer’ (sustaining proliferative signaling, evading growth suppressors, activating invasion and metastasis, enabling replicative immortality, inducing angiogenesis, and resistance to apoptotic signals) summarize the general characteristics of cells during tumor development [17]. miRNAs target protein-coding genes that have been approved for cancer therapeutics [18], suggesting that miRNAs play a major role in tumor development, including MM pathogenesis [19,20,21,22,23,24,25]. However, miRNA’s function in EMD pathogenesis remains unclear.

Currently, flow cytometry is a standard method for MM diagnosis. A combination of several antigens (CD38, CD138 and CD45) allows for the identification of the entire population of PCs in the BM. Since a normality assessment of PC is important as well, CD19 and CD56 are used as they can easily discriminate between normal PCs (CD19+CD56−) and abnormal PCs (CD19−CD56+/−). Abnormal PCs can be further characterized by several antigens (CD20, CD28, CD117 and CD200). For confirmation of PC clonality, cytoplasmic staining of kappa and lambda Ig chains is performed [26].

The aim of this study was to identify the miRNAs involved in the pathogenesis of EMD as well as the changes in cellular antigens during this process.

## 2. Results

### 2.1. Immunophenotypic Analysis of MM and EMD Patients

In total, samples of 70 MM and 31 EMD patients were analyzed. The expression levels of CD19, CD20, CD27, CD28, CD81, CD117 and nestin on abnormal PCs were low in both groups, although positive cases were detected. Using the nonparametric Mann–Whitney U-test, statistically significant differences between the MM and EMD patients were observed only in the case of clonal plasma cells (*p* = 0.011) (Table 1).

### 2.2. Small Non-Coding RNA Analysis

MiRNA analysis was performed as a two-step biomarker study. First, NGS was used in the exploration part of the study followed by validation by RT-qPCR.

#### 2.2.1. Next-Generation Sequencing

**Figure 1:**
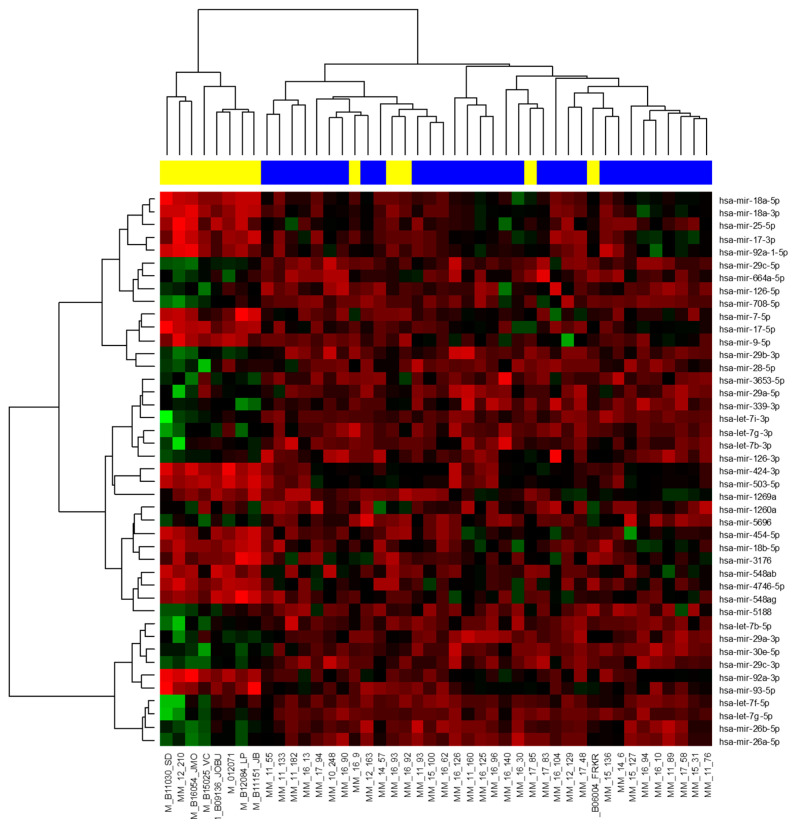
Heat map. A total of 43 miRNAs with significantly deregulated expression in patients with extramedullary disease (EMD, yellow) compared to multiple myeloma (MM) patients (blue) (adjusted *p* < 0.025).

In the exploration phase of the study, 44 small RNA libraries (31 MM vs. 13 EMD) were prepared and sequenced. The sequenced samples contained on average 8,817,426 reads, and 1,680,950 passed the filter. In total, 2278 different miRNAs were found to be present in the analyzed samples; 632 miRNAs had more than 20 reads per million in at least 20 samples and were included in subsequent analysis. From those, 43 miRNAs were identified as significantly deregulated (adjusted *p* < 0.025; Figure 1; Supplementary Table S1). Among the ten most deregulated miRNAs, five were found to be upregulated and five were downregulated in EMD patients compared to MM (Table 2).

#### 2.2.2. RT-qPCR Validation of MicroRNA

For the validation phase of the study, ten miRNAs (miR-18a-5p, miR-339-3p, miR-18a-3p, miR-92a 3p, miR-26a-5p, miR-29a-3p, miR-30e-5p, miR-424-3p, miR-126-3p and miR-708-5p) were chosen based on the results of NGS (|logFC| ≥ 1, average expression > 5, adjusted *p*-value < 0.05) and our previous preliminary data on a smaller cohort of patients [27]. First, the expression of all 10 miRNAs was analyzed in 26 MM and 10 EMD samples. Subsequently, the most promising miRNAs were chosen, and their expression was measured in the rest of samples (in total 39 MM and 20 EMD) by RT-qPCR. The average expression levels of all miRNAs were normalized using miR-107; this miRNA was chosen based on the NGS data as the most stably expressed miRNA and confirmed by GeNorm [28].

**Figure 2:**
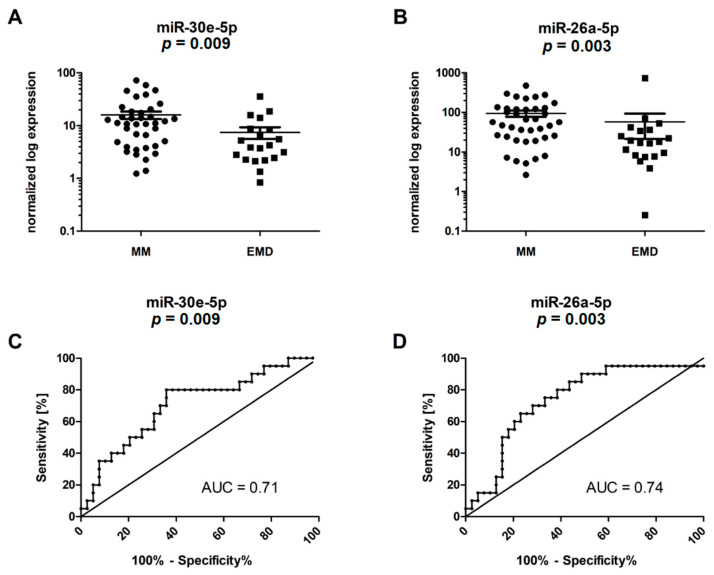
RT-qPCR validation and ROC analysis of selected microRNAs. (**A**) MiR-30e-5p was significantly downregulated in EMD vs. MM patients (*p* = 0.009). (**B**) MiR-26a-5p was significantly downregulated in EMD vs. MM patients (*p* = 0.003). (**C**) MiR-30e-5p enabled discrimination between MM and EMD patients (sensitivity 80.0%, specificity 64.1%; AUC = 0.71, cut-off = 8.8054). (**D**) MiR-26a-5p enabled discrimination between MM and EMD patients (sensitivity 80.0%, specificity 61.5%; AUC = 0.74, cut-off = 37.6610).

The RT-qPCR validation confirmed significantly upregulated levels of miR-30e-5p (*p* = 0.009) and miR-26a-5p (*p* = 0.003) in the MM compared to the EMD patients; other miRNAs were not statistically significantly deregulated (Table 3, Figure 2A,B). Subsequently, ROC analysis was performed to determine the capacity of individual miRNAs to distinguish the MM from the EMD patients. The best results were obtained for miR-26a-5p, which discriminated between these two groups of patients with a sensitivity of 80.0% and specificity of 61.5% (AUC = 0.74, cut off = 37.6610) (Figure 2C,D, Supplementary Table S2). Unfortunately, the combined analysis of miR-26a-5p and miR-30e-5p did not improve either the sensitivity or specificity of the test (AUC = 0.723, sensitivity 85%, specificity 62%).

### 2.3. Association between Respective Datasets and Correlation with Clinicopathological Data

Due to the low sample size in both the groups of patients and the availability of data (RT-qPCR, flow cytometry), the correlation of the parameters was assessed in the entire set of samples, regardless of EMD involvement. Table 4 describes the significantly correlated miRNA quantities with flow cytometry parameters.

Further, Spearman’s bivariate correlation was performed with continuous quantities to assess the correlation between miRNAs expression and the clinicopathological data of the patients. We observed a strong correlation between the levels of miR-26a-5p (rs = 0.274, *p* = 0.036) and miR-30e-5p (rs = 0.259, *p* = 0.048) and thrombocyte count (Supplementary Table S3).

A comparison between the miRNAs relative quantification and baseline characteristics was performed using the Mann–Whitney U-test with categorical variables. It revealed increased levels of miR-18a-5p (*p* = 0.033) and miR-26a-5p (*p* = 0.022) in women compared to men. Further, the expression of miR-18a-3p was significantly higher in patients with ISS stage III compared to stages I and II (*p* = 0.037), while significantly decreased levels of miR-92a-3p (*p* = 0.018), miR-424-3p (*p* = 0.023), miR-126-3p (*p* = 0.042) and miR-708-5p (*p* = 0.020) were found in patients with Durie–Salmon stage III compared to those with Durie–Salmon stage I or II. The correlations with other clinicopathological data of patients were not statistically significant (Supplementary Table S4).

### 2.4. Target Prediction and Protein Association Network Analysis

**Figure 3:**
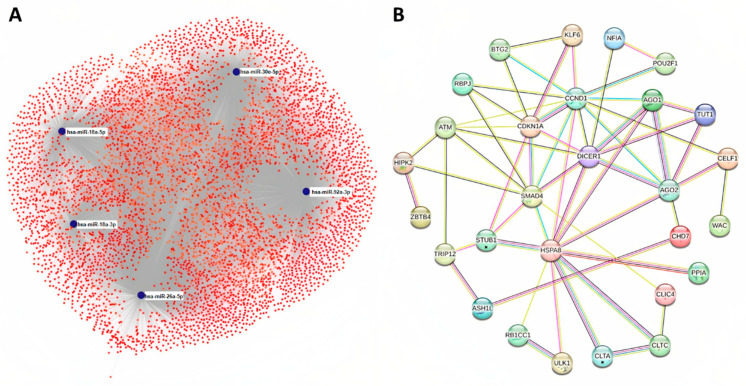
Target prediction and protein association network analysis. (**A**) In total, six thousand and thirty-seven different genes were predicted as potential targets of five significantly deregulated miRNAs (miR-18a-3p, miR-18a-5p, miR-26a-5p, miR-30e-5p and miR-92a) using miRNet online tool. (**B**) Protein–protein interaction network of twenty-six hub genes regulated by identified miRNAs (STRING) together with two other genes (with asterisk) significantly involved in the network. Light blue line—from curated databases, pink line—experimentally determined, yellow line—textmining, black line– co-expression, light purple line—protein homology, red line—gene fusions, dark blue line—gene co-occurrence.

The target genes of five significantly deregulated miRNAs (miR-18a-3p, miR-18a-5p, miR-26a-5p, miR-30e-5p and miR-92a-3p) were predicted by the miRNet online tool. In total, 6037 different genes were identified as depicted in Figure 3A. Further, miR-26a-5p was found to potentially target the higher number of predicted genes (3108 genes), followed by miR-92a-3p (2307 genes), miR-30e-5p (1617 genes), miR-18a-5p (1127 genes) and miR-18a-3p (649). Next, the protein–protein interaction networks of the predicted genes were constructed using the STRING database and Cytoscape software. Based on the degree and gene betweenness, the top 26 hub genes (together with CLTA and STUB1) were identified and included in the final network (Figure 3B, Supplementary Table S5). The strongest association was observed between RB1CC1/ULK1, AGO1/DICER1, AGO2/DICER and CCND1/CDKN1A (combined score 0.999; Supplementary Table S6).

### 2.5. Functional Annotation and Pathway Enrichment Analysis

**Figure 4:**
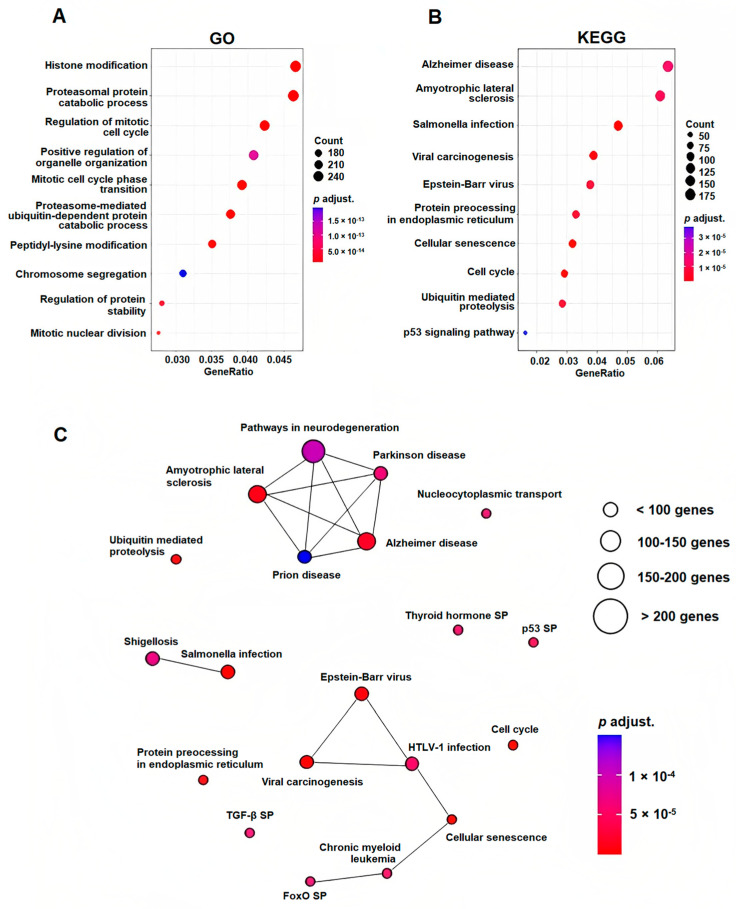
Functional annotation and pathway enrichment analysis. (**A**) Ten most enriched biological processes based on the GO classification. (**B**) Ten most enriched signaling pathways based on the KEGG analysis. (**C**) Signaling pathways interaction network based on the 20 most significant signaling pathways defined by KEGG. ALS—Amyotrophic lateral sclerosis, SP—signaling pathway, EB—Epstein–Barr virus, HTLV-1—Human T-cell leukemia virus 1, ER—endoplasmic reticulum, CML—chronic myeloid leukemia.

In order to analyze the involvement of the five significantly deregulated miRNAs and their target genes in biological processes and signaling pathways, GO and KEGG pathways enrichment analysis was performed. The most enriched pathways included cell cycle regulation and the p53 signaling pathway, ubiquitin-mediated proteolysis, protein processing in endoplasmic reticulum and cellular senescence. Further, there was significant enrichment in the pathways associated with viral carcinogenesis or bacterial infection and various neurological disorders. According to the GO classification of biological processes, the majority of genes were found to be involved in histone modification, the regulation of the mitotic cell cycle, proteasomal protein catabolic processes or positive regulation of organelle organization (Figure 4, Supplementary Table S7).

### 2.6. Overall Survival and Progression-Free Survival of MM and EMD Patients

The median length of follow up was 23.9 months (range: 0.3–126.9 months) in the MM and 13.9 months (range: 0.2–69.4 months) in the EMD patients. In the groups of 70 MM patients and 33 EMD patients, 41 MM (58.6%) and 24 EMD (72.7%) patients died.

**Figure 5:**
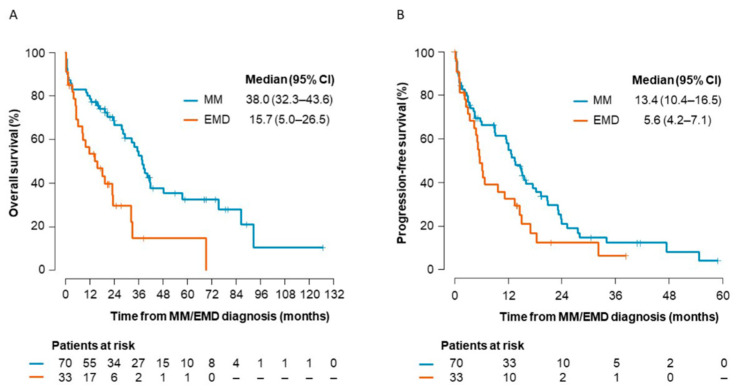
Overall survival and progression-free survival of multiple myeloma (MM) and extramedullary myeloma (EMD) patients. (**A**) MM patients had significantly longer overall survival compared to EMD patients (*p* < 0.001). (**B**) MM patients had significantly longer progression-free survival compared to EMD patients (*p* = 0.051).

Figure 5 shows the overall survival (OS) and progression-free survival (PFS) from the time of MM/EMD diagnosis, respectively. The median OS was 38.0 months (95% CI: 32.3–43.6) in MM and 15.7 months (95% CI: 5.0–26.5) in EMD (*p* < 0.001). The median PFS was 13.4 months (95% CI: 10.4–16.5) in MM and 5.6 months (95% CI: 4.2–7.1) in EMD (*p* = 0.051).

#### 2.6.1. Parameters Significantly Associated with PFS and OS of MM Patients

Concerning the clinicopathological data, a higher age (*p* < 0.001), clinical stage III vs. I (*p* = 0.001), clinical stage II vs. I (*p* = 0.020), decreased levels of albumin (*p* < 0.001), decreased levels of hemoglobin (*p* = 0.010), and increased levels of C-reactive protein (*p* = 0.001) and lactate dehydrogenase (*p* = 0.002) were associated with a significantly shorter PFS of MM patients, while a higher age (*p* < 0.001), clinical stage III vs. I (*p* = 0.033), other Ig vs. IgG (*p* = 0.039), decreased levels of albumin (*p* < 0.001), decreased levels of thrombocytes (*p* = 0.003), and increased levels of lactate dehydrogenase (*p* = 0.006) and C-reactive protein (*p* = 0.049) were detected in MM patients with a significantly shorter OS. In addition, a high expression of CD28 (*p* = 0.012) and the percentage of PCs in peripheral blood (*p* < 0.001) correlated with a shorter PFS, while increased levels of CD200 were associated with a worse overall survival of MM patients (*p* = 0.030; Supplementary Table S8). Finally, the correlation between miRNAs expression and the survival of MM patients was analyzed. Nevertheless, no significant association was found.

#### 2.6.2. Parameters significantly Associated with PFS and OS of EMD Patients

In EMD patients, clinical stage III vs. I (*p* = 0.014), Durie–Salmon substage B vs. A (*p* = 0.036), and increased levels of lactate dehydrogenase (*p* = 0.002) were significantly associated with a shorter PFS of EMD patients, while increased levels of IgA compared to IgG (*p* = 0.023) and high levels of beta-2-microglobulin (*p* = 0.018), lactate dehydrogenase (*p* = 0.002) and C-reactive protein (*p* = 0.012) were found in EMD patients with a significantly shorter OS. In addition, a significant correlation between the percentage of PCs in peripheral blood (*p* = 0.001 for both PFS and OS) as well as in bone marrow (*p* = 0.043 for OS) was confirmed (Supplementary Table S9). There was no correlation between the expression of the analyzed miRNAs and the PFS/OS of EMD patients.

## 3. Discussion

Multiple myeloma is the second most common hematological malignancy [1]. It is characterized by the infiltration of malignant PCs into the BM and the presence of monoclonal immunoglobulin in serum and/or urine. During the last 15 years, new drug protocols have increased the survival of MM patients dramatically [11]. At the same time, reports of a subset of MM, called extramedullary multiple myeloma (EMD), characterized by the migration of PCs into soft tissues, have increased. EMD is connected to a worse OS, PFS and a dire prognosis for the patients [6,7,8,11].

The pathogenesis of EMD has been at the center of attention of our clinical and research teams. First, our clinical study retrospectively analyzed 226 relapsed MM patients for the presence of EMD and found evidence in 24% of the relapsed MM patients. EMD occurred early in the course of the disease (53% at first relapse). The poorest outcome was found for the EMD-S when compared to the EMD-B patients (median OS 5 vs. 12 months; *p* = 0.006). Our data suggested that the complete independence of PCs from the BM leads to a poor outcome of the patients [6].

Next, we evaluated the common chromosomal aberrations of EMD patients using interphase fluorescence in situ hybridization. Overall, the frequency of all the studied chromosomal aberrations was the highest in the BM PCs of EMD patients. Our data showed that PCs gain more aberrations during their transformation into independent EMD PCs [29].

Our following study analyzed circulating miRNAs in the peripheral blood of EMD patients. We showed that circulating miR-130a may become a novel diagnostic marker of EMD [30].

Recently, we published a study suggesting that there are risk factors present already at the time of MM diagnosis that show a future development of secondary EMD, which should lead to a more careful follow-up for these patients [31].

In the present study, we concentrated on flow cytometric as well as miRNA analyses connected to the pathogenesis of EMD. Flow cytometric analysis has become one of the most relevant techniques for MM diagnosis as well as for the detection of minimal residual disease and risk assessment of MGUS progression [32,33,34]. While it is commonly used in MM diagnosis, no reports on the flow cytometric analysis of EMD have been published so far. We confirmed significantly higher levels of clonal PCs in the BM of EMD patients compared to patients with MM. Unfortunately, no significant changes in CD expression were observed between these two groups of samples. Further analysis of flow cytometry data confirmed a significant association between the expression of CD28 and CD200 on abnormal PCs and the survival of MM patients, while a worse prognosis of EMD patients was only associated with a higher percentage of PCs in peripheral blood and BM.

Similar results were observed previously, as CD28 is a key mediator of MM survival and apoptotic resistance [35], while increased levels of CD200 were observed in MM patients with a significantly shorter PFS and OS. Subsequent analysis of the dynamic changes in CD200 expression during treatment confirmed significantly lower levels of this antigen on the PCs of patients responding to therapy, and this decrease was associated with a favorable survival [36]. In addition, recent findings illustrate a clear correlation between the expression of CD200 on MM PCs and the frequency of immunosuppressive Treg cells involved in the enhancement of tumor growth and chemoresistance [37]. Altogether, these results indicate the possible use of CD200 as a novel prognostic and predictive biomarker in MM. 

MiRNAs are non-coding RNA molecules involved in the pathogenesis of various diseases, including MM [38]. Their role in EMD has not been clarified yet [27]. Thus, we concentrated on the deregulation of miRNAs in EMD using NGS and subsequent RT-qPCR validation. We identified miR-26a-5p and miR-30e-5p to be significantly upregulated in MM compared to EMD patients suggesting their potential role in EMD pathogenesis. In addition, based on the expression of these miRNAs, it was possible to discriminate between these two groups of patients with satisfactory sensitivity and specificity. Finally, elevated levels of miR-30e-5p and miR-18a-3p were associated with a poor prognosis of EMD patients. To the best of our knowledge, these miRNAs have not been described in EMD yet; however, their function was studied in different solid tumors as well as in MM. Hu et al. [38] found downregulated levels of miR-26a-5p in MM patients compared to healthy controls, and the expression of this miRNA correlated significantly with CD38 expression and therapeutic response to daratumumab. Using TaqMan Low Density miRNA Arrays, Jung et al. [39] identified six miRNAs, including miR-26a-5p, miR-30b-5p and miR-30c-5p, with significantly decreased levels in relapsed/refractory MM patients with a poor response to lenalidomide plus low-dose dexamethasone. Elevated levels of miR-30e-5p were observed in MM patients with a gain of 1q21 [40]. Concerning miR-18a, its low exosomal levels were previously associated with a poor survival of MM patients [41]. This miRNA is a component of the miR-17-92 cluster on chromosome 13q31.3, which is activated by the MYC proto-oncogene and is commonly amplified in various solid tumors and lymphomas and associated with a malignant progression of MM [24,42,43]. Further, downregulated levels of miR-18a were found in MM patients with retinoblastoma gene deletion [21]. Importantly, deregulation of the miR-17-92 cluster was not observed in MGUS patients compared to MM patients indicating the possible involvement of these miRNAs in tumor development and progression from MGUS to MM and EMD [24].

To better characterize the role of five significantly deregulated miRNAs in the pathogenesis of MM and EMD, their potential target genes were identified using the miRNet online tool. In total, more than 6000 genes were predicted, and approximately half of them were targeted by miR-26a-5p. Subsequently, the involvement of target genes in biological processes and signaling pathways was assessed using GO and KEGG pathways enrichment analysis. According to the GO classification, the majority of genes were found to be involved in histone modifications, regulation of the mitotic cell cycle and organelle organization or proteasomal protein catabolic processes. Based on the results of the KEGG analysis, several signaling pathways involved in cancerogenesis were confirmed to be influenced by the deregulated miRNAs, including cell cycle regulation, p53 signaling or cellular senescence. Interestingly, numerous target genes were associated with viral or bacterial infections as well as neurological disorders.

Infection is a major complication and the main cause of death of MM/EMD patients due to the immunodeficiency caused by the disease as well as the different treatment regiments. Importantly, the progression of disease from MM to EMD is commonly associated with cumulative immunosuppression and a higher rate of infections [44]. Thus, deregulated levels of miRNAs targeting the genes involved in viral cancerogenesis or bacterial infections may significantly contribute to the progression of MM into EMD. Nevertheless, it is not clear whether these changes are the cause or effect of various infections.

Myelomatous spread to the central nervous system (CNS) is a rare, but very serious, manifestation of MM, especially in patients with relapsed disease. In addition, various neurological disorders were proven to be subsequent complications of MM caused by both the disease and the treatment [45]. Further, degenerative CNS diseases such as Alzheimer’s disease as well as MM occur with increased frequency with advancing age [46] and are associated with increased levels of IL-6, which is a potent mediator of inflammation in both diseases. Interestingly, the down-regulation of this cytokine using estrogen or testosterone supplementation leads to effective management of MM and Alzheimer’s disease [47]. In addition, relatives of early-onset Alzheimer’s disease patients have an increased risk of lymphoreticular malignancies suggesting that the degenerative and demyelinating CNS diseases and the lymphoreticular malignancies may have a shared genetic profile, probably involving immunologic abnormality [46].

Previously, miR-26a was shown to suppress tumor growth and metastasis formation by targeting the IL-6/STAT3 pathway [48] and modulate the neuroinflammatory response induced by TLR4 stimulation [49]. On the other hand, the IL-6/STAT3 pathway increases miR-92a and miR-18a expression by directly targeting its promoter, which resulted in the activation of the Wnt/β-catenin signaling pathway and the promotion of the stem-like phenotype of cancer cells [50,51]. Several studies also proved the significant association between paraproteinemia and motor neuron diseases (MND), such as amyotrophic lateral sclerosis (ALS) [52]. It is not clear whether MM is directly involved in the pathogenesis of MND; however, it is possible that patients have some antibodies against motor neurons [53].

Finally, AA amyloidosis associated with the formation of insoluble amyloid fibrils depositing in various organs was detected in MM/EMD patients as well as patients with Alzheimer’s or Parkinson disease [54]. Importantly, the recent study of Rocanieres et al. [55] describes the role of the proteasome inhibitor ixazomib in the increased severity of Parkinson disease due to the defective function of the ubiquitin–proteasome system and protein elimination including misfolded amyloid proteins such as α-synuclein.

MiR-92a was previously confirmed to be significantly up-regulated in various cancers and to contribute to tumor progression by targeting the FBXW7 protein, which is a critical tumor suppressor and one of the most important molecules involved in the ubiquitin–proteasome system [56,57]. On the contrary, miR-30e-5p functions as an important inhibitor of cancerogenesis by targeting ubiquitin-specific peptidase 22 (USP22), which is a part of the SAGA complex involved in protein deubiquitination, cell cycle regulation and chromatin organization, and is highly expressed in brain tissue [58,59]. Altogether, these results indicate that down-regulated levels of miR-26a-5p and miR-30e-5p together with up-regulated levels of miRNAs from the miR-17-92 cluster may play an important role in the pathogenesis of EMD and its progression from MM. Nevertheless, further in vitro and in vivo functional studies will be necessary to confirm these assumptions.

Finally, a correlation between several CD antigens and the identified miRNAs was found, including a positive correlation between the expression of miR-30e-5p/miR-26a-5p/miR-92a-3p and CD81 positivity, while there was an inverse correlation between this antigen and the levels of miR-18a-3p. CD81 is a transmembrane protein from the tetraspanins family that is expressed on B-cells and plays a critical role in the activation of the B-cell receptor. While several in vitro studies proved the tumor-suppressive function of this molecule in MM cells, including reduced proliferation, migration and increased autophagy [60,61], its elevated levels in clinical samples were confirmed and associated with a worse prognosis [62]. Thus, the exact role of CD81 in MM progression into EMD is not clear yet.

Recently, Xu et al. [63] performed a meta-analysis to estimate the pooled hazard ratios for the correlations between miRNAs expression and the outcome of patients with MM. Interestingly, they proved that high levels of miR-92a are significantly associated with a poor prognosis and shorter survival.

Further, the positive correlation between the levels of miR-26a-5p/CD27 and miR-29a-3p/CD44 was proven, while a negative correlation was found in the case of miR-29a 3p/CD19. CD27 is a membrane glycoprotein of the TNF superfamily involved in the differentiation of B-cells into PCs. The loss of this antigen characterizes progression to MM, and its decreased levels were detected in patients with a shorter PFS and worse prognosis [64,65]. The role of CD27 in EMD is unknown; however, a low level of this molecule together with a significant down-regulation of miR-26a-5p were observed in this study.

CD44 is a cell surface adhesion receptor that is highly expressed in many cancers and regulates metastasis via the recruitment of CD44 to the cell surface. Its interaction with appropriate extracellular matrix ligands promotes the migration and invasion processes involved in the metastases of several solid tumors (breast, colon and gastric cancers) [66]. Its level was also upregulated on circulating PCs in MM [67]. Thus, it is probable that an increased expression of CD44 is connected to the migration of PCs out of the BM. Further, CD44 is an important marker of cancer stem cells, and several studies have previously highlighted the involvement of miR-29a-3p in cancer stem cell proliferation and differentiation [68,69].

CD19 is an important transmembrane protein that functions as an essential signaling component on the surface of mature B-cells. Previously, mRNA levels of this protein were found to correlate significantly with the expression of MYC-activated genes and were associated with a worse survival of lymphoma patients [70]. Importantly, the MYC-mediated downregulation of miR-29a-3p has been described in colorectal cancer and associated with an increased migration of tumor cells [71]. On contrary, this miRNA directly targets Smad nuclear interacting protein 1 (SNIP1), and its overexpression resulted in decreased migration, the proliferation of HeLa cells and lower expression levels of SNIP1 and its downstream genes including c-Myc [72]. Thus, the CD19/miR-29a-3p axis could be involved in the regulation of MYC-driven neoplastic growth of tumors derived from B-cells. The connection between other analyzed antigens and identified miRNAs has not been described so far.

All miRNAs and antigens identified in this study showed a significant role in the migration and survival of either MM cells directly or cancer cells in general. While our data were interesting, they need to be validated on a larger dataset of MM and EMD patients.

## 4. Materials and Methods

### 4.1. Patients’ Characteristics

In total, 103 patients (70 MM and 33 EMD) were involved in this study. For NGS analysis, we used 31 MM and 13 EMD patients; for RT-qPCR analysis, 39 MM and 20 EMD patients; and for flow cytometry, 70 MM patients and 31 EMD patients. Primary EMD was confirmed in 23 patients, while secondary EMD was detected in 10 patients. EMD diagnosis was based on imaging (X-ray, PET-CT, MRI, low-dose CT) and confirmed by the histopathology of biopsied samples. All MM patients’ samples were collected at the time of diagnosis (prior to any treatment). Clinical characteristics of patients are summarized in Supplementary Table S10. Treatment following MM and EMD diagnosis is included in Supplementary Table S11. All treatments before secondary EMD diagnosis are included in Supplementary Table S12.

As MM and EMD patients are rare, patients included in this study were diagnosed at the University Hospital Brno, University Hospital Olomouc and University Hospital Ostrava, Czech Republic, between the years 2006 and 2020. All patients signed informed consent approved by the Ethics committees of the hospitals in accordance with the current version of the Helsinki Declaration. This research was approved by the Ethics committee of the University Hospital Brno on 22/6/2016 (No. 21/2016).

### 4.2. Sample Preparation

Bone marrow PCs in mononuclear cell fraction were enriched by anti-CD138+ immunomagnetic beads using AutoMACS (Miltenyi Biotec, Bergisch Gladbach, Germany). Samples were frozen, stored at −80 °C and thawed only once. Only samples with a purity of CD38 + CD138 + PCs higher than 80% were used.

### 4.3. RNA Isolation

Total RNA enriched for small RNAs was isolated using an miRNeasy Mini Kit (Qiagen, Hilden, Germany) based on the manufacturer’s recommendations. Quality of isolated RNA was analyzed using an Agilent High Sensitivity RNA Screen Tape system (Agilent Technologies, Santa Clara, CA, USA). Concentration and purity of isolated RNA was measured on a NanoDrop 1000 Spectrophotometer. RNA Integrity number (RIN) was assessed by an Agilent High Sensitivity RNA Screen Tape System (Agilent Technologies, Germany) with a cut-off value of 7.

### 4.4. Next-Generation Sequencing

Next-generation sequencing (NGS) libraries were prepared using a CleanTagTM Small RNA Library Prep Kit (TriLink BioTechnologies, San Diego, CA, USA) according to the manufacturer’s instructions. A Qubit 2.0 Fluorometer (Thermo Fisher Scientific, Waltham, MA, USA) and an Agilent TapeStation System (Agilent, Santa Clara, CA, USA) were used for quality control of prepared libraries. A Pippin Prep (Sage Science, Beverly, MA, USA) was used for automatic size selection of the libraries before sequencing. Equimolar amounts of each cDNA library were pooled at a final concentration of 2 nmol·L^−1^. The sequencing run was performed on a NextSeq 500 (Illumina, San Diego, CA, USA) using a flow-cell with 50 bp single-end reads.

### 4.5. RT-qPCR Validation

For reverse transcription, a TaqMan^®^ Advanced miRNA cDNA Synthesis Kit was used (Applied Biosystems, Waltham, MA, USA) according to the manufacturer’s instructions with an RNA input of 5 ng. RT-qPCR was performed using TaqMan^®^ Advanced miRNA Assays (Applied Biosystems). All reactions were run in duplicates and incubated in a 96-well optical plate at 95 °C for 20 s followed by 40 cycles at 95 °C for 3 s and 60 °C for 30 s using a 7500 Real-Time PCR System (Applied Biosystems). The miRNA assay IDs are listed in Supplementary Table S13.

### 4.6. Flow Cytometry

Bone marrow samples were incubated with the following fluorescently labelled monoclonal antibodies (MoAbs): CD38-PB (Exbio, clone HIT2), CD45-PO (Exbio, clone HI30), CD28-FITC (Exbio, clone CD28.2), CD56-PE/APC (Exbio, clone LT56), CD138-PerCP (Exbio, clone MI15), CD117-APC (Exbio, clone 104D2), CD200-PE (Exbio, clone OX-104), CD19-PC7 (Beckman Coulter, clone J3-119), CD81-APC-H7 (Becton Dickinson, clone JS-81), CD44-APC-eFluor780 (eBioscience, clone IM7) CD27-APC-eFluor780/APC-AlexaFluor750 (eBioscience/Beckman Coulter, clones 470279/1A4CD27) and cytoplasmic nestin-APC (R&D, clone 196908). Eight color combinations of MoAbs were used for whole BM.

For only surface phenotyping, after 15 min of incubation with MoAb combinations, ammonium chloride was used for erythrolysis. For clonality assessment of PCs, cytoplasmic expression of immunoglobulin light chains was analyzed using Intraprep (Beckman Coulter) and polyclonal rabbit kappa-FITC/lambda-PE (Dako). Finally, samples were washed with PBS stabilized with sodium azide and then analyzed by a flow cytometer BD FACSCanto II (Becton Dickinson) equipped with three lasers (violet—405 nm, blue—488 nm, red—633 nm). Reanalysis of data was performed using the Infinicyt (Cytognos) software.

About 500,000 cells were analyzed for BM samples to obtain minimum of 5.000 CD38+CD138+ PCs per tube. Surface expression of CD19, CD20, CD27, CD28, CD44, CD56, CD81, CD117 and cytoplasmic nestin or kappa/lambda were analyzed on these PCs according to the recommendation of the European Myeloma Network [73]. PCs were considered positive for any given antigen if expression was over 20% [74]. Flow cytometric data were available for 101 (98%) patients.

### 4.7. Prediction of miRNAs Target Genes and Protein Association Network Analysis

Firstly, the target genes of five significantly deregulated miRNAs were predicted using miRNet 2.0 (http://www.mirnet.ca/ accessed on 4 February 2021), which is an easy-to-use comprehensive tool integrating data from three well-annotated databases miRTarBase v8.0, TarBase v8.0, and miRecords [75]. Subsequently, protein–protein interactions of target genes were analyzed using the Search Tool for the Retrieval of Interacting Genes/Proteins (STRING) v11.5 (https://string-db.org/ accessed on 10 November 2022) and visualized with the Cytoscape 3.9.1 software [76]. Finally, gene ontology (GO) and Kyoto Encyclopedia of Genes and Genomes (KEGG) pathways enrichment analysis was performed in order to analyze the involvement of selected genes in biological processes and signaling pathways using the R/Bioconductor packages multiMiR and GDCRNATools [77].

### 4.8. Statistical Analysis

Count-based miRNA expression data were generated by the Chimira tool from fastq files. All sequences were adapter-trimmed and mapped against miRBase v20 allowing up to two mismatches per sequence. Further analyses were performed using the R/Bioconductor packages. MiRNAs having less than twenty reads per samples were dropped out. The read counts were pre-normalized by adding normalization factors within the edgeR package and further between sample normalized by the voom function in the LIMMA package. After the normalized expression levels were determined, the differentially expressed miRNAs between EMD patients and MM patients were screened applying linear model fitting and the eBayes approach. The obtained *p*-values were adjusted for multiple testing using the Benjamini–Hochberg method.

In RT-qPCR analysis, the threshold cycle (CT) values of the analyzed genes were obtained using the 7500 SDS Software version 1.4.0 (Applied Biosystems). The average expression levels of miRNAs were normalized using miR-107 as it was the most stably expressed miRNA based on the results of NGS analysis and it was subsequently verified by GeNorm [28]. Further, their relative expression was calculated by the 2^−ΔCT^ method. Statistical differences between MM and EMD samples were analyzed using the nonparametric Mann–Whitney U-test. Further, ROC analysis was performed to obtain sensitivity and specificity values for each individual miRNA. *p*-values below 0.05 were considered as statistically significant. All calculations from the validation phase of the study were carried out using GraphPad Prism 8 (GraphPad Software, San Diego, CA, USA).

Data were described by the absolute and relative frequencies of categorical variables and median (minimum–maximum) of quantitative variables. Differences between groups of patients were tested using Fisher’s exact test and the Mann–Whitney U-test. Correlation in continuous parameters was assessed using Spearman’s correlation coefficient. OS and PFS from time of MM/EM diagnosis (date of sample collection) was plotted using the Kaplan–Meier methodology. The log–rank test was used to estimate the statistical significance of the difference between the curves. Cox’s proportional hazards model was performed to explore the univariate association of risk factors with OS and PFS. Cut-off values for parameters significantly associated with OS/PFS were defined using time-dependent ROC analysis (concerning parameters from flow cytometry analysis or miRNA quantity). *p*-values less than 0.05 were considered statistically significant (all tests were two-sided). Analysis was performed in the SPSS software (IBM Corp. Released 2017. IBM SPSS Statistics for Windows, Version 25.0., IBM Corp.: Armonk, NY, USA) and R software version 3.3.0 (www.r-project.org).

## Figures and Tables

**Table 1:** Flow cytometry analysis at diagnosis of multiple myeloma (MM) and extramedullary myeloma (EMD) patients. Median of relative expression on abnormal plasma cells (A-PCs) is shown (%).

Plasma Cells Phenotype	MM (*n* = 70)		EMD (*n* = 31)		*p* ^1^
	*n*	Median (Min–Max)	*n*	Median (Min–Max)	
**% PC—peripheral blood**	*n* = 70	0.1 (0.0–13.2)	*n* = 31	0.1 (0.0–7.0)	0.074
**% PC—bone marrow**	*n* = 70	21.0 (1.2–64.5)	*n* = 31	17.6 (0.9–72.5)	0.222
**CD19 A-PC**	*n* = 70	0.0 (0.0–55.6)	*n* = 31	0.0 (0.0–8.0)	0.221
**CD56 A-PC**	*n* = 70	99.1 (0.0–100.0)	*n* = 31	99.6 (0.0–100.0)	0.660
**CD20 A-PC**	*n* = 39	0.0 (0.0–91.7)	*n* = 20	0.0 (0.0–22.6)	0.965
**CD27 A-PC**	*n* = 69	2.3 (0.0–99.0)	*n* = 31	3.8 (0.0–98.0)	0.994
**CD28 A-PC**	*n* = 67	0.0 (0.0–99.5)	*n* = 26	0.1 (0.0–99.7)	0.219
**CD44 A-PC**	*n* = 65	66.7 (0.0–100.0)	*n* = 25	92.5 (0.9–100.0)	0.290
**CD81 A-PC**	*n* = 61	2.0 (0.0–98.5)	*n* = 27	2.0 (0.0–99.2)	0.914
**CD117 A-PC**	*n* = 68	3.4 (0.0–99.7)	*n* = 28	0.1 (0.0–99.3)	0.082
**CD200 A-PC**	*n* = 54	97.5 (0.0–100.0)	*n* = 20	85.2 (0.0–100.0)	0.184
**nestin A-PC**	*n* = 61	0.9 (0.0–100.0)	*n* = 22	9.8 (0.0–98.2)	0.204
**aPC**	*n* = 67	99.9 (98.0–100.0)	*n* = 31	99.6 (72.7–100.0)	**0.011**

^1^ Mann–Whitney U-test. A-PC—abnormal plasma cells; aPC—clonal plasma cells. Statistically significant values are highlighted in bold.

**Table 2:** List of ten most deregulated microRNAs (miRNAs) between the patients with extramedullary disease (EMD) and multiple myeloma (MM) patients identified during the exploration phase of the study by next-generation sequencing.

miRNA	logFC	Average Expression	*p*-Value	Adjusted *p*-Value
miR-4746-5p	1.891	0.467	2.432 · 10^−6^	1.185 · 10^−3^
**miR-26a-5p**	−1.157	16.334	3.751 · 10^−6^	1.185 · 10^−3^
**miR-92a-3p**	1.693	13.075	9.052 · 10^−6^	1.328 · 10^−3^
miR-548ag	2.029	0.789	1.155 · 10^−5^	1.328 · 10^−3^
**miR-18a-3p**	1.759	5.093	1.700 · 10^−5^	1.328 · 10^−3^
**miR-339-3p**	−1.403	5.490	1.723 · 10^−5^	1.328 · 10^−3^
miR-3653-5p	−1.717	4.819	1.806 · 10^−5^	1.328 · 10^−3^
**miR-30e-5p**	−1.096	12.501	2.003 · 10^−5^	1.328 · 10^−3^
**miR-29a-3p**	−1.367	12.986	2.100 · 10^−5^	1.328 · 10^−3^
**miR-18a-5p**	1.872	5.292	2.101 · 10^−5^	1.328 · 10^−3^

FC—fold change; miRNAs in bold were chosen for further validation phase of the study.

**Table 3:** Normalized expression levels of selected microRNAs (miRNAs) in validation phase of the study.

miRNA		MM (*n* = 39)		EMD (*n* = 20)	*p* ^1^
	*n*	Median (Min–Max)	*n*	Median (Min–Max)	
**miR-18a-5p**	*n* = 39	0.76 (0.02–12.37)	*n* = 20	0.72 (0.00–3.50)	0.706
**miR-18a-3p**	*n* = 39	0.00 (0.00–0.39)	*n* = 20	0.01 (0.00–1.29)	0.318
**miR-30e-5p**	*n* = 39	11.08 (1.23–72.24)	*n* = 20	4.06 (0.84–35.96)	**0.009**
**miR-92a-3p**	*n* = 39	8.27 (0.97–91.61)	*n* = 20	5.10 (1.04–34.61)	0.163
**miR-26a-5p**	*n* = 39	56.81 (2.64–476.74)	*n* = 20	17.61 (0.25–741.21)	**0.003**
miR-29a-3p	*n* = 26	20.93 (4.20–161.13)	*n* = 10	34.38 (8.45–176.17)	0.741
miR-424-3p	*n* = 26	0.03 (0.00–3.90)	*n* = 10	0.07 (0.00–28.35)	0.539
miR-126-3p	*n* = 26	3.01 (0.03–29.64)	*n* = 10	1.09 (0.01–22.50)	0.189
miR-339-3p	*n* = 26	0.43 (0.15–3.83)	*n* = 10	0.59 (0.00–4.19)	0.475
miR-708-5p	*n* = 26	0.11 (0.00–2.59)	*n* = 10	0.14 (0.00–3.19)	0.590

^1^ Mann–Whitney U-test, *p*-values in bold are statistically significant, miRNAs in bold were measured in all samples involved in the analysis.

**Table 4:** Correlation of microRNA (miRNA) quantity and flow cytometry parameters.

miRNA	Flow-Cytometry	*n*	*r_S_*	*p* ^1^
**miR-18a-5p**	% PC—peripheral blood	59	0.278	**0.033**
	nestin A-PC	53	0.347	**0.011**
**miR-339-3p**	CD27 A-PC	35	0.303	0.077
**miR-18a-3p**	% PC—peripheral blood	59	0.267	**0.041**
	CD81 A-PC	54	−0.278	**0.042**
	CD200 A-PC	52	−0.254	0.069
**miR-30e-5p**	CD20 A-PC	59	0.221	0.092
	CD27 A-PC	58	0.226	0.088
	CD81 A-PC	54	0.362	**0.007**
**miR-92a-3p**	CD81 A-PC	54	0.351	**0.009**
**miR-26a-5p**	CD27 A-PC	58	0.312	**0.017**
	CD81 A-PC	54	0.384	**0.004**
	aPC	59	0.218	0.097
**miR-29a-3p**	% PC—bone marrow	36	−0.295	0.081
	CD19 A-PC	36	−0.381	**0.022**
	CD44 A-PC	33	0.407	**0.019**
	CD117 A-PC	32	0.310	0.084
**miR-424-3p**	CD20 A-PC	36	−0.422	**0.010**
	CD81 A-PC	32	0.342	0.056
	aPC	36	−0.293	0.082
**miR-126-3p**	CD117 A-PC	32	0.440	**0.012**
**miR-339-3p**	CD27 A-PC	35	0.303	0.077
**miR-708-5p**	CD20 A-PC	36	−0.282	0.096
	CD44 A-PC	33	0.330	0.061

^1^ Correlation of miRNA quantity and flow cytometry parameters with *p* < 0.1 reported. rs correlation coefficient, A-PC—abnormal plasma cells, aPC—clonal plasma cells.

## Data Availability

NGS data are accessible at https://www.ncbi.nlm.nih.gov/geo/query/acc.cgi?acc=GSE129918, access code knghsugetxebjar. After publication, the data will become publicly available.

## Supplementary Materials

Supporting information can be downloaded at: https://www.mdpi.com/article/10.3390/ijms232314765/s1, Tables S1–S6. **Supplementary Table S1**: A list of 43 significantly deregulated microRNAs (miRNA) between patients with extramedullary disease (EMD) and multiple myeloma (MM) patients (adjusted *p* < 0.025) identified in the exploration phase of the study by next-generation sequencing; **Supplementary Table S2**: Receiver operating characteristic analysis of validated microRNAs; **Supplementary Table S3**: Spearman’s bivariate correlation between the expression of microRNAs (miRNAs) and continuous clinicopathological data of patients with multiple myeloma (MM) and extramedullary disease (EMD); **Supplementary Table S4**: Correlation between the expression of microRNAs (miRNAs) and categorical clinical characteristics of patients with multiple myeloma (MM) and extramedullary disease (EMD) using Mann–Whitney U-test or Kruskal–Wallis test; **Supplementary Table S5**: Cox’s proportional hazards model—association of clinical parameters and flow-cytometry parameters with overall survival (OS) and progression-free survival (PFS) of multiple myeloma (MM) patients; **Supplementary Table S6**: Protein–protein interaction network of 28 hub genes regulated by key miRNAs significantly deregulated in patients with extramedullary disease compared to multiple myeloma; **Supplementary Table S7**: Signaling pathways and biological processes associated with deregulated expression of miR-18a-5p, miR-18a-3p, miR-92a-3p, miR-30e-5p and miR-26a-5p and their target genes; **Supplementary Table S8**: Cox’s proportional hazards model—association of clinical parameters and flow-cytometry parameters with overall survival (OS) and progression-free survival (PFS) of multiple myeloma (MM) patients; **Supplementary Table S9**: Cox’s proportional hazards model—association of clinical parameters and flow-cytometry parameters with overall survival (OS) and progression-free survival (PFS) of extramedullary disease patients (EMD) patients; **Supplementary Table S10**: Clinical characteristics of patients involved in the study at the time of disease diagnosis; **Supplementary Table S11**: Treatment in the first line after extramedullary disease (EMD) or multiple myeloma (MM) diagnosis; **Supplementary Table S12**: Previous therapy in any treatment line before secondary extramedullary disease (EMD) diagnosis; **Supplementary Table S13**: The IDs of microRNAs assays used in validation phase of the study.
