# Supplementary material for: Involvement of Small Non-Coding RNA and Cell Antigens in Pathogenesis of Extramedullary Multiple Myeloma
Source: Int J Mol Sci. 2022 Nov 25;23(23):14765. doi: 10.3390/ijms232314765 (PMC9741227; doi:10.3390/ijms232314765)
Supplement: Supplementary file 1 [file ijms-23-14765-s001.zip › ijms-2057207-supplementary.pdf]

Involvement of small non-coding RNA and cell antigens in pathogenesis of extramedullary multiple myeloma

Supplementary Table S1 A list of 43 significantly deregulated microRNAs (miRNA) between patients with extramedullary disease (EMD) and multiple myeloma (MM) patients (adjusted $p < 0.025$) identified in the exploration phase of the study by next-generation sequencing.

**miRNA**	**logFC**	**average expression**	***p*-value**	**adjusted *p*-value**
miR-4746-5p	1.891	0.467	$2.432 \cdot 10^{-6}$	$1.185 \cdot 10^{-3}$
**miR-26a-5p**	-1.158	16.334	$3.751 \cdot 10^{-6}$	$1.185 \cdot 10^{-3}$
**miR-92a-3p**	1.693	13.075	$9.052 \cdot 10^{-6}$	$1.328 \cdot 10^{-3}$
miR-548ag	2.029	0.789	$1.155 \cdot 10^{-5}$	$1.328 \cdot 10^{-3}$
**miR-18a-3p**	1.759	5.093	$1.700 \cdot 10^{-6}$	$1.328 \cdot 10^{-3}$
**miR-339-3p**	-1.403	5.490	$1.723 \cdot 10^{-5}$	$1.328 \cdot 10^{-3}$
miR-3653-5p	-1.717	4.819	$1.806 \cdot 10^{-5}$	$1.328 \cdot 10^{-3}$
**miR-30e-5p**	-1.096	12.501	$2.003 \cdot 10^{-5}$	$1.328 \cdot 10^{-3}$
**miR-29a-3p**	-1.367	12.986	$2.100 \cdot 10^{-5}$	$1.328 \cdot 10^{-3}$
**miR-18a-5p**	1.872	5.292	$2.101 \cdot 10^{-5}$	$1.328 \cdot 10^{-3}$
let-7b-5p	-1.497	13.210	$2.797 \cdot 10^{-5}$	$1.423 \cdot 10^{-3}$
let-7i-3p	-1.955	5.201	$3.060 \cdot 10^{-5}$	$1.423 \cdot 10^{-3}$
miR-26b-5p	-0.914	15.080	$3.149 \cdot 10^{-5}$	$1.423 \cdot 10^{-3}$
miR-9-5p	1.933	10.263	$3.367 \cdot 10^{-5}$	$1.423 \cdot 10^{-3}$
**miR-126-3p**	-2.066	4.211	$3.599 \cdot 10^{-5}$	$1.423 \cdot 10^{-3}$
miR-29c-5p	-1.275	7.227	$3.602 \cdot 10^{-5}$	$1.423 \cdot 10^{-3}$
let-7g-5p	-1.112	15.376	$3.920 \cdot 10^{-5}$	$1.457 \cdot 10^{-3}$
miR-29b-3p	-1.197	10.088	$6.843 \cdot 10^{-5}$	$2.403 \cdot 10^{-3}$
miR-29c-3p	-1.336	11.945	$7.586 \cdot 10^{-5}$	$2.523 \cdot 10^{-3}$
let-7g-3p	-1.308	3.750	$1.802 \cdot 10^{-4}$	$5.694 \cdot 10^{-3}$
miR-548ab	1.618	0.500	$2.019 \cdot 10^{-4}$	$6.077 \cdot 10^{-3}$
**miR-708-5p**	-2.726	6.888	$4.810 \cdot 10^{-4}$	$1.382 \cdot 10^{-2}$
miR-25-5p	1.129	5.989	$5.079 \cdot 10^{-4}$	$1.396 \cdot 10^{-2}$
miR-29a-5p	-1.250	4.769	$6.020 \cdot 10^{-4}$	$1.585 \cdot 10^{-2}$
miR-1269a	3.690	3.895	$6.778 \cdot 10^{-4}$	$1.713 \cdot 10^{-2}$
**miR-424-3p**	2.652	4.654	$7.321 \cdot 10^{-4}$	$1.780 \cdot 10^{-2}$
miR-3176	1.426	1.564	$8.029 \cdot 10^{-4}$	$1.824 \cdot 10^{-2}$
miR-28-5p	-1.373	8.471	$8.419 \cdot 10^{-4}$	$1.824 \cdot 10^{-2}$
miR-5696	-1.133	2.076	$8.455 \cdot 10^{-4}$	$1.824 \cdot 10^{-2}$
miR-17-3p	1.083	7.981	$8.657 \cdot 10^{-4}$	$1.824 \cdot 10^{-2}$
miR-1260a	-1.135	2.420	$1.047 \cdot 10^{-3}$	$2.074 \cdot 10^{-2}$
miR-454-5p	1.003	3.645	$1.079 \cdot 10^{-3}$	$2.074 \cdot 10^{-2}$
miR-503-5p	3.300	3.735	$1.088 \cdot 10^{-3}$	$2.074 \cdot 10^{-2}$
miR-17-5p	1.167	10.283	$1.116 \cdot 10^{-3}$	$2.074 \cdot 10^{-2}$
miR-93-5p	0.906	12.228	$1.172 \cdot 10^{-3}$	$2.099 \cdot 10^{-2}$
let-7b-3p	-1.335	3.250	$1.196 \cdot 10^{-3}$	$2.099 \cdot 10^{-2}$
miR-664a-5p	-1.103	6.126	$1.264 \cdot 10^{-3}$	$2.159 \cdot 10^{-2}$
miR-126-5p	-1.507	6.056	$1.323 \cdot 10^{-3}$	$2.201 \cdot 10^{-2}$

miR-18b-5p	1.685	1.953	$1.530 \cdot 10^{-3}$	$2.455 \cdot 10^{-2}$
miR-5188	-1.222	0.339	$1.593 \cdot 10^{-3}$	$2.455 \cdot 10^{-2}$
miR-92a-1-5p	1.358	7.485	$1.651 \cdot 10^{-3}$	$2.468 \cdot 10^{-2}$
miR-7-5p	1.243	10.751	$1.679 \cdot 10^{-3}$	$2.468 \cdot 10^{-2}$
miR-106a-5p	1.133	5.876	$2.064 \cdot 10^{-3}$	$2.899 \cdot 10^{-2}$

FC – fold change; miRNAs in bold were chosen for further validation phase of the study

Supplementary Table S2 Receiver operating characteristic analysis of validated microRNAs.

EMD vs. MM	AUC (95% CI)	p-value	cut-off	Sensitivity	Specificity
**miR-18a-5p**	0.53 (0.38–0.69)	0.701	–		
**miR-339-3p**	0.58 (0.38–0.79)	0.458	–		
**miR-18a-3p**	0.58 (0.43–0.73)	0.313	–		
**miR-30e-5p**	0.71 (0.57–0.85)	**0.009**	≤8.8054	80.0%	64.1%
**miR-92a-3p**	0.61 (0.46–0.76)	0.159	–		
**miR-26a-5p**	0.74 (0.60–0.87)	**0.003**	≤37.6610	80.0%	61.5%
**miR-29a-3p**	0.54 (0.34–0.74)	0.724	–		
**miR-424-3p**	0.57 (0.36–0.78)	0.525			
**miR-126-3p**	0.65 (0.43–0.86)	0.180			
**miR-708-5p**	0.56 (0.35–0.78)	0.572			

[1]AUC – area under the curve, *p*-values in bold are statistically significant.

Supplementary Table S3 Spearman bivariate correlation between the expression of microRNAs (miRNAs) and continuous clinicopathological data of multiple myeloma (MM) and extramedullary disease (EMD) patients.

**miRNA**	**Clinical characteristics**	**N**	r_S	***p***
**miR-339-3p**	Age	36	-0.307	0.068
**miR-18a-3p**	Serum M-protein quantity ($g \cdot l^{-1}$)	57	-0.251	0.060
**miR-30e-5p**	Thrombocyte count ($10^9 \cdot l^{-1}$)	59	0.259	**0.048**
**miR-92a-3p**	CRP ($mg \cdot l^{-1}$)	59	-0.222	0.091
**miR-26a-5p**	Thrombocyte count ($10^9 \cdot l^{-1}$)	59	0.274	**0.036**
**miR-708-5p**	Age	36	-0.313	0.063

[1] Correlation of miRNA quantity and clinical characteristics with $p<0.1$ reported. *p*-values in bold are statistically significant.

Supplementary Table S4 Correlation between the expression of microRNAs (miRNAs) and categorical clinical characteristics of patients with multiple myeloma (MM) and extramedullary disease (EMD) using Mann-Whitney U-test or Kruskal-Wallis test.

**Clinical characteristics**	**miR-18a-5p**		**miR-339-3p**		**miR-18a-3p**		**miR-92a-3p**		**miR-26a-5p**		**miR-29a-3p**		**miR-424-3p**		**miR-126-3p**		**miR-708-5p**	
	**n**	**median (min–max)**	**n**	**median (min–max)**	**n**	**median (min–max)**	**n**	**median (min–max)**	**n**	**median (min–max)**	**n**	**median (min–max)**	**n**	**median (min–max)**	**n**	**median (min–max)**	**n**	**median (min–max)**
**Sex**																		
women	24	1.13 (0.00–12.37)	17	0.79 (0.20–4.19)	–	–	–	–	24	59.58 (2.64–741.21)	–	–	17	–	17	3.63 (0.12–29.64)	–	–
men	35	0.51 (0.00–2.89)	19	0.35 (0.00–2.18)	–	–	–	–	35	25.84 (0.25–278.54)	–	–	19	–	19	1.23 (0.01–29.62)	–	–
p-value		0.033		0.073		n. s.		n. s.		0.022		n. s.		n. s.		0.090		n. s.
**ISS**																		
Stage 1	–	–	–	–	18	0.01 (0.00–0.39)	–	–	–	–	9	68.86 (14.19–176.17)	–	–	–	–	–	–
Stage 2	–	–	–	–	21	0.00 (0.00–0.05)	–	–	–	–	16	19.41 (4.20–60.46)	–	–	–	–	–	–
Stage 3	–	–	–	–	20	0.01 (0.00–1.29)	–	–	–	–	11	22.77 (7.26–161.13)	–	–	–	–	–	–
p-value		n. s.		n. s.		0.037		n. s.		n. s.		0.084		n. s.		n. s.		n. s.
**Durie-Salmon stage**																		
I, II	–	–	3	1.14 (1.03–2.18)	–	–	6	14.13 (7.94–61.74)	–	–	–	–	3	1.28 (0.43–28.35)	3	22.50 (3.47–29.62)	3	0.76 (0.33–3.19)
III	–	–	33	0.44 (0.00–4.19)	–	–	53	6.05 (0.97–91.61)	–	–	–	–	33	0.03 (0.00–3.90)	33	1.56 (0.01–29.64)	33	0.09 (0.00–2.59)
p-value		n. s.		0.071		n. s.		0.018		n. s.		n. s.		0.023		0.042		0.020
**Durie-Salmon substage**																		
A	–	–	–	–	–	–	–	–	–	–	–	–	–	–	27	3.47 (0.01–29.64)	–	–
B	–	–	–	–	–	–	–	–	–	–	–	–	–	–	9	0.88 (0.03–8.49)	–	–
p-value		n. s.		n. s.		n. s.		n. s.		n. s.		n. s.		n. s.		0.060		n. s.
**M-protein type**																		
IgG	–	–	–	–	–	–	–	–	–	–	–	–	–	–	–	–	24	0.12 (0.01–3.19)
IgA	–	–	–	–	–	–	–	–	–	–	–	–	–	–	–	–	8	0.05 (0.00–0.41)
other	–	–	–	–	–	–	–	–	–	–	–	–	–	–	–	–	4	0.26 (0.11–0.54)
p-value		n. s.		n. s.		n. s.		n. s.		n. s.		n. s.		n. s.		n. s.		0.058

Supplementary Table S5: A set of 26 hub genes regulated by significantly deregulated miRNAs (miR-18a-3p, miR-18a-5p, miR-92a-3p, miR-26a-5p, miR-30e-5p) and potentially involved in the pathogenesis of multiple myeloma and extramedullary disease.

Gene symbol	Degree	Betweenness	Gene symbol	Degree	Betweenness
ASH1L	5	24435.75	NFIA	4	16999.39
ATM	5	24435.75	CHD7	4	16999.39
CELF1	5	24435.75	BTG2	4	16028.45
DICER1	5	24435.75	CDKN1A	4	16028.45
GAN	5	24435.75	CLIC4	4	16028.45
HIPK2	5	24435.75	PPIA	4	16028.45
KLHDC10	5	24435.75	SMAD4	4	16028.45
KLHL15	5	24435.75	TUT1	4	16028.45
NUCKS1	5	24435.75	ZBTB4	4	16028.45
RBPJ	5	24435.75	HSPA8	4	16028.45
WAC	5	24435.75	KLF6	4	16028.45
AGO1	4	16999.39	POU2F1	4	16028.45
TRIP12	4	16999.39	ULK1	4	16028.45

Supplementary Table S6: Protein-protein interaction network of 28 hub genes regulated by key miRNAs significantly deregulated in patients with extramedullary disease compared to multiple myeloma.

PPI	String db combined score	String db textmining	String db databases	String db experiments	String db co-expression
RB1CC1-ULK1	0.999	0.995	0.900	0.951	0.062
AGO2-DICER1	0.999	0.994	0.900	0.978	0.068
CCND1-CDKN1A	0.999	0.990	0.900	0.983	0.085
AGO1-DICER1	0.999	0.994	0.900	0.940	0.068
CCND1-HSPA8	0.986	0.973	-	0.502	-
SMAD4-CDKN1A	0.981	0.528	0.900	0.632	-
AGO2-AGO1	0.976	0.058	0.900	0.769	0.064
CCND1-SMAD4	0.958	0.602	0.900	-	-
HSPA8-CLTC	0.954	0.831	0.600	0.324	0.116
CCND1-BTG2	0.943	0.459	0.900	-	-
CCND1-POU2F1	0.910	0.123	0.900	-	0.062
SMAD4-HSPA8	0.904	0.081	0.900	-	-
BTG2-KLF6	0.782	0.377	-	-	0.666
AGO2-CCND1	0.781	0.402	0.650	-	-
AGO2-HSPA8	0.773	0.680	-	0.305	0.062
CCND1-AGO1	0.720	0.234	0.650	-	-
CCND1-ATM	0.712	0.712	-	-	-
TRIP12-ASH1L	0.707	0.589	-	0.078	0.290
ATM-CDKN1A	0.702	0.703	-	-	-
TUT1-DICER1	0.684	0.626	-	0.142	0.095
SMAD4-RBPJ	0.640	0.626	-	-	0.076
ATM-SMAD4	0.639	0.626	-	-	0.076
HSPA8-DICER1	0.553	0.372	-	0.290	0.078
ATM-HPK2	0.551	0.542	-	-	0.062
CCND1-KLF6	0.541	0.534	-	0.056	-
RBPJ-CDKN1A	0.532	0.522	-	-	0.062
PPIA-HSPA8	0.529	0.398	-	0.166	0.137
AGO2-CHD7	0.518	0.455	-	-	0.151
CCND1-DICER1	0.517	0.512	-	-	0.052
CDKN1A-DICER1	0.516	0.407	-	0.218	-
ZBTB4-HIPK2	0.512	0.286	-	0.316	0.082
SMAD4-DICER1	0.506	0.449	-	0.121	0.063
WAC-CELF1	0.505	0.495	-	-	0.062
ATM-TRIP12	0.499	0.410	-	-	0.186
BTG2-CDKN1A	0.496	0.458	-	-	0.110
AGO2-TUT1	0.490	0.401	-	0.179	0.048
CLIC4-CLTC	0.490	0.464	-	-	0.086
ATM-DICER1	0.484	0.456	-	-	0.092
CCND1-RBPJ	0.481	0.469	-	-	0.062
POU2F1-NFIA	0.469	0.455	-	0.067	-
TUT1-AGO1	0.455	0.360	-	0.179	0.048
SMAD4-CLIC4	0.453	0.453	-	-	-
ASH1L-CHD7	0.453	0.306	-	0.153	0.144
AGO2-CELF1	0.451	0.368	-	0.128	0.085
AGO1-HSPA8	0.435	0.158	-	0.346	0.057
SMAD4-HIPK2	0.432	0.418	-	-	0.064
ULK1-HSPA8	0.426	0.416	-	0.058	-
CCND1-CELF1	0.423	0.410	-	-	0.062
CDKN1A-KLF6	0.409	0.370	-	0.058	0.085
RB1CC1-HSPA8	0.406	0.406	-	-	-
NFIA-DICER1	0.403	0.390	-	-	0.062

PPI – protein-protein interaction

Supplementary Table S7 Signaling pathways and biological processes associated with deregulated expression of miR-18a-5p, miR-18a-3p, miR-92a-3p, miR-30e-5p, and miR-26a-5p and their target genes.

Category	Description	Term name	Gene ratio	Bg ratio	FDR value	*P*-value
KEGG pathway	Cell cycle	hsa04110	81/2784	126/8190	$8.88 \cdot 10^{-10}$	$2.67 \cdot 10^{-12}$
KEGG pathway	Salmonella infection	hsa05132	131/2784	249/8190	$1.14 \cdot 10^{-7}$	$6.86 \cdot 10^{-10}$
KEGG pathway	Cellular senescence	hsa04218	89/2784	156/8190	$2.48 \cdot 10^{-7}$	$2.24 \cdot 10^{-9}$
KEGG pathway	Viral carcinogenesis	hsa05203	108/2784	204/8190	$1.18 \cdot 10^{-6}$	$1.41 \cdot 10^{-8}$
KEGG pathway	Protein processing in endoplasmic reticulum	hsa04141	92/2784	171/8190	$3.80 \cdot 10^{-6}$	$6.14 \cdot 10^{-8}$
KEGG pathway	Epstein-Barr virus infection	hsa05169	105/2784	202/8190	$3.80 \cdot 10^{-6}$	$7.83 \cdot 10^{-8}$
KEGG pathway	Ubiquitin mediated proteolysis	hsa04120	79/2784	142/8190	$3.80 \cdot 10^{-6}$	$7.99 \cdot 10^{-8}$
KEGG pathway	Amyotrophic lateral sclerosis	hsa05014	170/2784	364/8190	$8.18 \cdot 10^{-6}$	$1.96 \cdot 10^{-7}$
KEGG pathway	Alzheimer disease	hsa05010	177/2784	384/8190	$1.23 \cdot 10^{-5}$	$3.33 \cdot 10^{-7}$
KEGG pathway	p53 signaling pathway	hsa04115	45/2784	73/8190	$3.82 \cdot 10^{-5}$	$1.15 \cdot 10^{-6}$
KEGG pathway	Human T-cell leukemia virus 1 infection	hsa05166	109/2784	222/8190	$5.08 \cdot 10^{-5}$	$1.80 \cdot 10^{-6}$
KEGG pathway	Chronic myeloid leukemia	hsa05220	46/2784	76/8190	$5.08 \cdot 10^{-5}$	$1.83 \cdot 10^{-6}$
KEGG pathway	Thyroid hormone signaling pathway	hsa04919	66/2784	121/8190	$5.70 \cdot 10^{-5}$	$2.37 \cdot 10^{-6}$
KEGG pathway	TGF-beta signaling pathway	hsa04350	54/2784	94/8190	$5.70 \cdot 10^{-5}$	$2.40 \cdot 10^{-6}$
KEGG pathway	Nucleocytoplasmic transport	hsa03013	60/2784	108/8190	$6.26 \cdot 10^{-5}$	$3.06 \cdot 10^{-6}$
KEGG pathway	Parkinson disease	hsa05012	126/2784	266/8190	$6.26 \cdot 10^{-5}$	$3.15 \cdot 10^{-6}$
KEGG pathway	FoxO signaling pathway	hsa04068	70/2784	131/8190	$6.26 \cdot 10^{-5}$	$3.19 \cdot 10^{-6}$
KEGG pathway	Shigellosis	hsa05131	118/2784	247/8190	$7.13 \cdot 10^{-5}$	$3.85 \cdot 10^{-6}$
KEGG pathway	Pathways of neurodegeneration - multiple diseases	hsa05022	207/2784	476/8190	$1.02 \cdot 10^{-4}$	$5.83 \cdot 10^{-6}$
KEGG pathway	Prion disease	hsa05020	127/2784	273/8190	$1.49 \cdot 10^{-4}$	$8.95 \cdot 10^{-6}$
GO biological process	histone modification	GO:0016570	269/5766	494/18800	$5.98 \cdot 10^{-25}$	$9.40 \cdot 10^{-29}$
GO biological process	proteasomal protein catabolic process	GO:0010498	267/5766	496/18800	$5.32 \cdot 10^{-24}$	$1.67 \cdot 10^{-27}$
GO biological process	regulation of mitotic cell cycle	GO:0007346	244/5766	478/18800	$9.45 \cdot 10^{-18}$	$4.45 \cdot 10^{-21}$
GO biological process	proteasome-mediated ubiquitin-dependent protein catabolic process	GO:0043161	217/5766	414/18800	$1.82 \cdot 10^{-17}$	$1.14 \cdot 10^{-20}$
GO biological process	mitotic cell cycle phase transition	GO:0044772	226/5766	440/18800	$6.19 \cdot 10^{-17}$	$4.86 \cdot 10^{-20}$
GO biological process	peptidyl-lysine modification	GO:0018205	202/5766	392/18800	$3.19 \cdot 10^{-15}$	$3.01 \cdot 10^{-18}$

GO biological process	regulation of protein stability	GO:0031647	162/5766	300/18800	$2.06 \cdot 10^{-14}$	$2.41 \cdot 10^{-17}$
GO biological process	mitotic nuclear division	GO:0140014	159/5766	293/18800	$2.06 \cdot 10^{-14}$	$2.59 \cdot 10^{-17}$
GO biological process	positive regulation of organelle organization	GO:0010638	235/5766	487/18800	$1.08 \cdot 10^{-13}$	$1.53 \cdot 10^{-16}$
GO biological process	chromosome segregation	GO:0007059	179/5766	348/18800	$1.97 \cdot 10^{-13}$	$3.21 \cdot 10^{-16}$
GO biological process	sister chromatid segregation	GO:0000819	119/5766	205/18800	$1.97 \cdot 10^{-13}$	$3.41 \cdot 10^{-16}$
GO biological process	regulation of DNA metabolic process	GO:0051052	226/5766	472/18800	$9.26 \cdot 10^{-13}$	$1.74 \cdot 10^{-15}$
GO biological process	DNA replication	GO:0006260	149/5766	280/18800	$1.19 \cdot 10^{-12}$	$2.43 \cdot 10^{-15}$
GO biological process	spindle organization	GO:0007051	109/5766	188/18800	$3.03 \cdot 10^{-12}$	$6.67 \cdot 10^{-15}$
GO biological process	organelle fission	GO:0048285	232/5766	493/18800	$3.52 \cdot 10^{-12}$	$8.30 \cdot 10^{-15}$
GO biological process	peptidyl-lysine acetylation	GO:0018394	107/5766	184/18800	$3.54 \cdot 10^{-12}$	$8.90 \cdot 10^{-15}$
GO biological process	regulation of cell cycle phase transition	GO:1901987	201/5766	415/18800	$5.65 \cdot 10^{-12}$	$1.53 \cdot 10^{-14}$
GO biological process	nuclear division	GO:0000280	213/5766	446/18800	$5.65 \cdot 10^{-12}$	$1.60 \cdot 10^{-14}$
GO biological process	internal peptidyl-lysine acetylation	GO:0018393	101/5766	173/18800	$1.17 \cdot 10^{-11}$	$3.51 \cdot 10^{-14}$
GO biological process	positive regulation of cellular catabolic process	GO:0031331	213/5766	449/18800	$1.17 \cdot 10^{-11}$	$3.81 \cdot 10^{-14}$

Bg – background, FDR – false discovery rate

Supplementary Table S8 Cox proportional hazards model - association of clinical parameters and flow-cytometry parameters with overall survival (OS) and progression free survival (PFS) of multiple myeloma (MM) patients.

Characteristics at MM diagnosis (N = 70)	Overall survival (OS)		Progression-free survival (PFS)	
	HR (95% CI)	*p*[1]	HR (95% CI)	*p*[1]
**Clinical characteristics**				
ISS 1 - reference	–	–	–	–
ISS stage 2 vs. stage 1	2.53 (0.92–6.94)	0.071	2.88 (1.18–7.01)	**0.020**
ISS stage 3 vs. stage 1	2.93 (1.09–7.87)	**0.033**	4.03 (1.74–9.30)	**0.001**
IgG - reference	–	–	–	–
IgA vs. IgG	1.18 (0.54–2.60)	0.675	0.73 (0.37–1.46)	0.378
Other vs. IgG	0.22 (0.05–0.93)	**0.039**	0.49 (0.21–1.13)	0.095
Age	1.10 (1.06–1.14)	**<0.001**	1.08 (1.04–1.12)	**<0.001**
Hemoglobin level ($g{\cdot}l^{-1}$)	0.99 (0.97–1.01)	0.196	0.98 (0.96–1.00)	**0.010**
Thrombocyte count ($10^9{\cdot}l^{-1}$)	0.99 (0.99–1.00)	**0.003**	1.00 (0.99–1.00)	0.113
Calcium total level ($mmol{\cdot}l^{-1}$)	0.32 (0.10–1.10)	0.071	0.44 (0.16–1.24)	0.121
Albumin level ($g{\cdot}l^{-1}$)	0.89 (0.84–0.94)	**<0.001**	0.91 (0.87–0.96)	**<0.001**
Beta-2-microglobulin ($mg{\cdot}l^{-1}$)	1.03 (1.00–1.05)	0.051	1.02 (1.00–1.04)	0.081
LDH ($\mu kat{\cdot}l^{-1}$)	1.35 (1.09–1.68)	**0.006**	1.40 (1.13–1.73)	**0.002**
CRP ($mg{\cdot}l^{-1}$)	1.01 (1.00–1.02)	**0.049**	1.01 (1.01–1.02)	**0.001**
**Flow-cytometry**				
% PC – peripheral blood	1.09 (0.95–1.25)	0.219	1.24 (1.11–1.39)	**0.000**
CD28 A-PC	1.01 (0.99–1.02)	0.387	1.01 (1.00–1.02)	**0.012**
CD200 A-PC	1.01 (1.00–1.03)	**0.030**	1.00 (0.99–1.01)	0.984

[1] *p*-values smaller than 0.1 reported

Supplementary Table S9 Cox proportional hazards model - association of clinical parameters and flow-cytometry parameters with overall survival (OS) and progression free survival (PFS) of extramedullary disease patients (EMD) patients.

Characteristics at EMD diagnosis (N = 33)	Overall survival (OS)		Progression-free survival (PFS)	
	**HR (95% CI)**	***p*[1]**	**HR (95% CI)**	***p*[1]**
**Clinical characteristics**				
ISS 1 - reference	–	–	–	–
ISS stage 2 vs. stage 1	1.07 (0.37–3.10)	0.903	2.61 (0.91–7.51)	0.075
ISS stage 3 vs. stage 1	2.43 (0.88–6.73)	0.088	3.98 (1.32–11.96)	**0.014**
Substage A - reference	–	–	–	–
Durie-Salmon substage B vs. A	2.64 (0.98–7.09)	0.054	2.67 (1.07–6.67)	**0.036**
IgG - reference	–	–	–	–
IgA vs. IgG	0.32 (0.12–0.85)	**0.023**	0.56 (0.24–1.33)	0.190
Other vs. IgG	0.60 (0.17–2.15)	0.436	1.49 (0.52–4.29)	0.464
Creatinine level ($\mu mol \cdot l^{-1}$)	1.00 (1.00–1.01)	0.059	1.00 (1.00–1.01)	0.066
Beta-2-microglobulin ($mg \cdot l^{-1}$)	1.07 (1.01–1.13)	**0.018**	1.04 (0.99–1.10)	0.091
LDH ($\mu kat \cdot l^{-1}$)	1.12 (1.04–1.19)	**0.002**	1.12 (1.04–1.21)	**0.002**
CRP ($mg \cdot l^{-1}$)	1.02 (1.01–1.04)	**0.012**	1.02 (1.00–1.03)	0.052
**miRNA[#]**				
miR-18a-3p	1.22 (0.98–1.52)	0.075	1.21 (0.97–1.51)	0.093
miR-30e-5p	1.01 (1.00–1.02)	0.095	1.01 (1.00–1.01)	0.057
**Flow-cytometry**				
% PC – peripheral blood	1.87 (1.30–2.68)	**0.001**	1.90 (1.31–2.75)	**0.001**
% PC – bone marrow	1.02 (1.00–1.04)	**0.043**	1.01 (0.99–1.03)	0.259
CD28 A-PC	1.00 (0.99–1.02)	0.771	1.01 (1.00–1.02)	0.051

[1] *p*-values smaller than 0.1 reported; [#]HR describes association of length of OS/PFS with 0.1 increase of respective miRNA.

Supplementary Table S10 Clinical characteristics of patients involved in the study at the time of disease diagnosis.

**Basic characteristics at diagnosis**[1]		**MM (N = 70)**	**EMD (N = 33)**	p^2
**Sex**	woman	36 (51.4%)	7 (21.2%)	**0.005**
	man	34 (48.6%)	26 (78.8%)	
**Age**	≤60	15 (21.4%)	11 (33.3%)	
	61–70	25 (35.7%)	6 (18.2%)	0.269
	71–80	25 (35.7%)	14 (42.4%)	
	>80	5 (7.1%)	2 (6.1%)	
	median (min–max)	68.5 (40.0–89.0)	69.0 (44.0–82.0)	0.593
**Follow-up (months)**	median (min–max)	23.9 (0.3–126.9)	13.9 (0.2–69.4)	**0.001**
**ECOG**	0	16 (22.9%)	1 (3.0%)	
	1	26 (37.1%)	14 (42.4%)	**0.045**
	2	19 (27.1%)	10 (30.3%)	
	3–4	9 (12.9%)	8 (24.2%)	
**ISS**	stage 1	16 (22.9%)	11 (33.3%)	
	stage 2	26 (37.1%)	9 (27.3%)	0.462
	stage 3	28 (40.0%)	13 (39.4%)	
**Durie-Salmon stage**	I	1 (1.4%)	0 (0.0%)	
	II	10 (14.3%)	2 (6.1%)	0.544
	III	59 (84.3%)	31 (93.9%)	
**Durie-Salmon substage**	A	48 (68.6%)	25 (75.8%)	0.496
	B	22 (31.4%)	8 (24.2%)	
**M-protein type**	IgG	47 (67.1%)	14 (42.4%)	
	IgA	13 (18.6%)	13 (39.4%)	**0.020**
	LC only	10 (14.3%)	4 (12.1%)	
	IgM	0 (0.0%)	1 (3.0%)	
	IgD	0 (0.0%)	1 (3.0%)	
**Serum M-protein quantity (g·l^{-1}) (N = 99)**	median (min–max)	33.0 (0.0–97.9)	16.1 (0.0–80.8)	**0.012**
**Light chain type**	kappa	47 (67.1%)	15 (45.5%)	0.052
	lambda	23 (32.9%)	18 (54.5%)	
**Hemoglobin level (g·l^{-1})**	median (min–max)	96.1 (69.0–149.0)	109.0 (69.0–149.0)	**0.029**
**Thrombocyte count (10^9·l^{-1})**	median (min–max)	225.5 (89.0–493.0)	182.0 (67.0–473.0)	0.263
**Calcium total level (mmol·l^{-1})**	median (min–max)	2.4 (1.6–3.9)	2.4 (1.9–3.4)	0.796
**Albumin level (g·l^{-1})**	median (min–max)	35.2 (15.0–49.8)	37.2 (25.6–47.2)	0.300
**Creatinine level (µmol·l^{-1})**	median (min–max)	102.5 (45.0–1346.0)	95.0 (46.0–540.0)	0.638
**Beta-2-microglobulin (mg·l^{-1}) (N = 101)**	median (min–max)	4.7 (1.9–50.0)	3.6 (1.7–27.4)	0.356
**LDH (µkat·l^{-1}) (N = 102)**	median (min–max)	3.3 (1.4–9.9)	3.7 (2.3–35.2)	**0.025**
**CRP (mg·l^{-1}) (N = 102)**	median (min–max)	5.7 (0.0–136.0)	6.3 (0.0–99.6)	0.459
**Plasmocyte count (%) – bone marrow cytology (N = 100)**	median (min–max)	39.9 (2.8–88.4)	33.9 (3.6–81.6)	0.3548

[1]Described using N (%) in categorical variables and median (minimum–maximum) in continuous variables.
[2]*p*-value of Fisher's exact test in categorical variables and Mann-Whitney *U* test in continuous variables.
MM – multiple myeloma, EMD – extramedullary disease, ISS – international staging system, LDH – lactate dehydrogenase, CRP – C-reactive protein

Supplementary Table S11 Treatment in the first line after extramedullary disease (EMD) or multiple myeloma (MM) diagnosis.

**Treatment regimen in first line after MM / EMD diagnosis**	**MM (N = 70)**	**EMD (N = 33)**
**bortezomib** + cyclophosphamide + dexamethasone	22 (31.4%)	10 (30.3%)
**bortezomib** + doxorubicin + dexamethasone	11 (15.7%)	4 (12.1%)
**bortezomib** + **thalidomide** + dexamethasone	7 (10.0%)	3 (9.1%)
**bortezomib** + dexamethasone	7 (10.0%)	2 (6.1%)
**bortezomib** + **lenalidomide** + dexamethasone	7 (10.0%)	1 (3.0%)
**bortezomib** + **thalidomide** + cyclophosphamide + dexamethasone	4 (5.7%)	2 (6.1%)
salvage autologous transplantation	–	2 (6.1%)
**lenalidomide** + doxorubicin + dexamethasone	–	2 (6.1%)
**bortezomib** + prednisone	2 (2.9%)	–
**bortezomib** + melphalan + prednisone	2 (2.9%)	–
**lenalidomide** + **daratumumab** + dexamethasone	–	1 (3.0%)
**lenalidomide** + **carfilzomib** + dexamethasone	–	1 (3.0%)
**daratumumab** + dexamethasone + melflufen	–	1 (3.0%)
**carfilzomib** + melphalan + prednisone	1 (1.4%)	–
**bortezomib** + **thalidomide** + doxorubicin + dexamethasone	1 (1.4%)	–
**bortezomib** + melphalan + dexamethasone	–	1 (3.0%)
**bortezomib** + melphalan + **daratumumab** + prednisone	1 (1.4%)	–
**bortezomib** + **lenalidomide** + methylprednisolon	–	1 (3.0%)
**bortezomib** + **lenalidomide** + dexamethasone + **isatuximab**	1 (1.4%)	–
**bortezomib** + cyclophosphamide + prednisone	–	1 (3.0%)
**bortezomib** + cyclophosphamide + **daratumumab** + dexamethasone	1 (1.4%)	–
**bortezomib** + cyclophosphamide	1 (1.4%)	–
Without treatment (patient died)	2 (2.9%)	1 (3.0%)
Transplantation	21 (30.0%)	7 (21.2%)

Supplementary Table S12 Previous therapy in any treatment line before secondary extramedullary disease (EMD) diagnosis.

**Previous therapy of patients diagnosed with secondary EMD (N = 10 patients)**	**N (%)**
**Proteasome inhibitors (PI)**	9 (90.0%)
bortezomib	8 (80.0%)
carfilzomib	1 (10.0%)
**Immunomodulatory drugs (IMiD)**	6 (60.0%)
lenalidomide	4 (40.0%)
thalidomide	3 (30.0%)
**Corticosteroids**	10 (100.0%)
dexamethasone	9 (90.0%)
prednisone	1 (10.0%)
**Conventional chemotherapy**	8 (80.0%)
bendamustine	1 (10.0%)
cyclophosphamide	5 (50.0%)
doxorubicin	5 (50.0%)
vincristine	1 (10.0%)
**Transplantation**	5 (50.0%)

Supplementary Table S13 The IDs of microRNAs assays used in validation phase of the study.

miRNA	Assay ID
**hsa-miR-18a-5p**	478551_mir
**hsa-miR-339-3p**	478325_mir
**hsa-miR-18a-3p**	477944_mir
**hsa-miR-92a-3p**	477827_mir
**hsa-miR-424-3p**	478091_mir
**hsa-miR-29a-3p**	478587_mir
**hsa-miR-126-3p**	477887_mir
**hsa-miR-26a-5p**	477995_mir
**hsa-miR-708-5p**	478197_mir
**hsa-miR-30e-5p**	478388_mir
**hsa-miR-107**	478254_mir
